# Muscle Architecture Adaptations to Static Stretching Training: A Systematic Review with Meta-Analysis

**DOI:** 10.1186/s40798-023-00591-7

**Published:** 2023-06-15

**Authors:** Ioli Panidi, Olyvia Donti, Andreas Konrad, Petros C. Dinas, Gerasimos Terzis, Athanasios Mouratidis, Vasiliki Gaspari, Anastasia Donti, Gregory C. Bogdanis

**Affiliations:** 1 School of Physical Education and Sport Science, National and Kapodistrian University of Athens, Dafne, Greece; 2 Institute of Human Movement Science, Sport and Health, University of Graz, Graz, Austria; 3 FAME Laboratory, Department of Physical Education and Sport Science, University of Thessaly, Trikala, Greece

**Keywords:** Fascicle length, Muscle thickness, Pennation angle, Cross-sectional area, Stretching, Ultrasound

## Abstract

**Background:**

Long-term stretching of human skeletal muscles increases joint range of motion through altered stretch perception and decreased resistance to stretch. There is also some evidence that stretching induces changes in muscle morphology. However, research is limited and inconclusive.

**Objective:**

To examine the effect of static stretching training on muscle architecture (i.e., fascicle length and fascicle angle, muscle thickness and cross-sectional area) in healthy participants.

**Design:**

Systematic review and meta-analysis.

**Methods:**

PubMed Central, Web of Science, Scopus, and SPORTDiscus were searched. Randomized controlled trials and controlled trials without randomization were included. No restrictions on language or date of publication were applied. Risk of bias was assessed using Cochrane RoB2 and ROBINS-I tools. Subgroup analyses and random-effects meta-regressions were also performed using total stretching volume and intensity as covariates. Quality of evidence was determined by GRADE analysis.

**Results:**

From the 2946 records retrieved, 19 studies were included in the systematic review and meta-analysis (n = 467 participants). Risk of bias was low in 83.9% of all criteria. Confidence in cumulative evidence was high. Stretching training induces trivial increases in fascicle length at rest (SMD = 0.17; 95% CI 0.01–0.33; *p* = 0.042) and small increases in fascicle length during stretching (SMD = 0.39; 95% CI 0.05 to 0.74; *p* = 0.026). No increases were observed in fascicle angle or muscle thickness (*p* = 0.30 and *p* = 0.18, respectively). Subgroup analyses showed that fascicle length increased when high stretching volumes were used (*p* < 0.004), while no changes were found for low stretching volumes (*p* = 0.60; subgroup difference: *p* = 0.025). High stretching intensities induced fascicle length increases (*p* < 0.006), while low stretching intensities did not have an effect (*p* = 0.72; subgroup difference: *p* = 0.042). Also, high intensity stretching resulted in increased muscle thickness (*p* = 0.021). Meta-regression analyses showed that longitudinal fascicle growth was positively associated with stretching volume (*p* < 0.02) and intensity (*p* < 0.04).

**Conclusions:**

Static stretching training increases fascicle length at rest and during stretching in healthy participants. High, but not low, stretching volumes and intensities induce longitudinal fascicle growth, while high stretching intensities result in increased muscle thickness.

***Registration*:**

PROSPERO, registration number: CRD42021289884.

**Supplementary Information:**

The online version contains supplementary material available at 10.1186/s40798-023-00591-7.

## Key Points

- Static stretching training induces trivial increases in fascicle length at rest and small increases in fascicle length during stretching.
- High, but not low, stretching volumes and intensities induce longitudinal fascicle growth.
- High stretching intensities result in increased muscle thickness.
- Fascicle angle remains unaffected by static stretching training.

**Supplementary Information:**

The online version contains supplementary material available at 10.1186/s40798-023-00591-7.

## Background

Human skeletal muscle responds to mechanical loading by adapting its structure [1]. Muscle structure can be described by quantifying its architectural parameters, namely fascicle length and angle, muscle thickness, and cross-sectional area, using ultrasonography [2, 3]. Mechanical loading induced either by muscle contraction or muscle stretching triggers alterations in cellular signaling and gene expression, which modify the physiological, structural, and contractile properties of muscle fibers [1, 4, 5]. Training using lengthening vs. shortening muscle contractions, leads to greater increases in strength, fascicle length and cross-sectional area [6]. On the other hand, stretch-induced mechanical tension has been shown to increase fascicle length, muscle mass, mean fiber thickness, and fiber number in animals [7, 8], but the volumes and intensities of such interventions are very different from what is typically applied in humans.

Skeletal muscle stretching is commonly used in sports and clinical settings, with the aim to increase maximum joint range of motion (ROM) and muscle-tendon unit extensibility [9]. Increased ROM following long-term stretching interventions may be explained by increased stretch tolerance [10] and/or changes in tissue mechanical properties [11–13], while some recent studies have found changes in muscle morphology [14, 15]. To date, however, muscle architectural adaptations to static stretching in humans are unclear [9, 16–19]. Most studies found no detectable changes in fascicle angles and muscle thickness following static stretching training [9, 14, 15, 17, 19], with some notable exceptions [15, 16, 20–22]. Regarding fascicle length, an increase in resting values has been found following 6–12 weeks of stretching training [9, 14, 23], while increases in muscle fascicle length during stretching may appear earlier, i.e., after 3–4 weeks of static stretching training [24, 25]. In contrast, other studies did not detect changes in muscle architecture following stretching interventions [24, 26]. For example, in an earlier meta-analysis examining the effects of three types of stretching training on joint mechanical properties [19] no increases in fascicle length were found following 2–8 weeks of training. The authors assumed that the three stretching types may target different the tissues around a joint e.g., PNF stretching may target tendon stiffness more than static stretching since the contraction during stretch overstretches the tendon [27]. Thus, the conflicting results between studies can be partly attributed to differences in stretching protocols and methodologies used [19]. Longer-term static stretching interventions [9, 15], overloaded static stretching [14], and high-intensity and/or long-duration stretching bouts [15, 23] may be more effective in inducing changes in muscle morphology.

Collectively, there seems to be no consensus on the feasibility and magnitude of muscle architectural changes after stretching training in humans, as well as on the stretching load characteristics required to induce changes in muscle morphology [14, 17, 24, 26]. Since changes in muscle architecture are linked to muscle contractile properties in healthy participants (e.g., force and power generation) [28, 29] and clinical populations [30], it would be of great interest to examine the potential adaptations of muscle architecture to static stretching. Therefore, the current systematic review aimed to examine the effects of static stretching training on muscle architecture (fascicle length and fascicle angle, muscle thickness, and cross-sectional area) and to conduct a meta-analysis. In addition, we examined if stretch-induced adaptations in muscle architecture are dependent on stretch volume and intensity.

## Methods

This systematic review was conducted according to the Preferred Reporting Items for Systematic Reviews and Meta-Analyses (PRISMA) guidelines [31] (see Additional file 1: S1 for PRISMA checklist). The review was preregistered with the International Prospective Register of Systematic Reviews (PROSPERO; registration number: CRD42021289884).

### Search and Selection Strategy

PICOS (Population, Intervention, Comparison, Outcome, Study Design) was used to form the research question and to select the search terms. Four reviewers searched independently four electronic databases (IP, VG, AD, OD): PubMed Central, Scopus, Web of Science, and SPORTDiscus to identify studies examining the effect of static stretching training on muscle architecture (i.e., fascicle length and fascicle angle, muscle thickness and cross-sectional area). The search was completed in July 2022 and the keywords used in the above databases are reported in the Additional file 2: S2. No language, study design and date restrictions were applied in the search algorithm. The field types used in the search were: “Title”, “Abstract” and “Keywords”. Additional records were found by: (1) searching the reference lists of relevant review papers and studies meeting the eligibility criteria (2) screening the researchers’ personal lists (first authors) in ResearchGate and Google Scholar [32, 33]. Furthermore, two studies which were not identified in the systematic searches were also included in the meta-analysis, based on our knowledge of the area. Three investigators (AD, AK and PCD) selected the eligible studies, and disagreements were resolved by GCB and GT by majority consensus. Reliability of study selection was calculated using the Kappa agreement coefficient, which was between 0.747 and 0.836.

### Inclusion and Exclusion Criteria

Randomized controlled trials (RCTs) and controlled trials without randomization (CTs) using static stretching training lasting ≥ 3 weeks were included. The limit of 3 weeks was chosen according to the relevant literature, as the shortest stretching training intervention of the eligible studies. Studies with healthy (i.e., non-clinical), recreationally active or trained participants were included. Comparisons were made between delta values (i.e., post- minus pre-intervention measurements) of experimental and control groups. Studies with the following characteristics were excluded: (a) studies examining the acute effects of static stretching, (b) studies combining static stretching with other interventions, such as strength training, etc., (c) studies examining very small joints, such as fingers, (d) animal or in vitro studies, (e) review papers, retrospective studies, case reports, letters to the editor, special communications, invited commentaries and conference papers.

### Risk of Bias Assessment and Methodological Quality

IP and OD independently assessed the risk of bias (RoB) of the included studies, and any conflict was resolved through discussion with GCB and AK. Risk of bias for randomized controlled trials and controlled trials without randomization was assessed using the updated Cochrane Risk of Bias 2 (RoB 2) and Risk of Bias in Non-randomized Studies-of Interventions (ROBINS-I**)**, respectively. The sources of bias included in the updated Risk of Bias 2 (RoB2) Cochrane library were: bias arising from the randomization process, bias due to deviations from intended interventions (effect of assignment to intervention and effect of adhering to intervention), bias due to missing outcome data, bias in the measurement of the outcome, and bias in selection of the reported result [34]. The sources of bias included in ROBINS-I were: bias due to confounding, bias in selection of participants into the study, bias in classification of interventions, bias due to deviations from intended interventions, bias due to missing data, bias in measurement of outcomes, and bias in selection of the reported results [34, 35]. 

### Confidence in Cumulative Evidence

Quality and confidence in the cumulative evidence were assessed using the Grading of Recommendations, Assessment, Development and Evaluations (GRADE) quality rating analysis. GRADE includes four levels of evidence quality: very low, low, moderate, and high [35, 36]. For randomized controlled trials, GRADE starts by assuming high quality, which can be downgraded according to five evaluation components (Risk of Bias, Inconsistency of results, Indirectness, Imprecision and Publication Bias) [35, 36], while three evaluation components were used to upgrade quality (Large Effect, Dose Response, Confounding). GRADE analysis was performed independently by IP and OD and was verified by GCB and PCD.

### Data Extraction

Data extraction from the included papers was performed by three independent investigators (IP, VG, and AK), and was supervised by two referee investigators (GT and PCD). The following data fields were extracted: (a) authors, (b) date and type of publication (journal, paper or grey literature), (c) study design type (RCT or CT) (d) sample size, sex and age of the experimental and control groups, (e) anthropometric characteristics of the experimental and control groups (body mass and height) (f) physical activity level of the participants (g) main outcomes of the study (means and standard deviations) regarding fascicle length (at rest and during stretching), fascicle angle and muscle thickness for the experimental and control groups. Cross-sectional area of the gastrocnemius muscle was measured in only two studies [15, 37] and thus a meta-analysis could not be performed. The results of these two studies are briefly reported in the Discussion. The characteristics of the included studies can be found in Table 1.

**Table 1:** Characteristics and main outcomes of the included studies

Study	Study design	Total participants (n)	Males	Females	SG (n)	CG (n)	Age (SG)	Age (CG)	Participants' physical activity	Architectrural characteristics	Main outcome
Akagi and Takahashi [42]	RCT	19	19	**-**	Unilateral design		23.7 ± 2.3		Sedentary or recreationally active participants	TH	A 5-week unilateral stretching intervention decreased gastrocnemius muscle hardness but did not change muscle thickness and the ratio of GM hardness to GL hardness
Andrade et al. [9]	RCT	39	19	20	21	18	21.0 ± 2.4	21.1 ± 2.0	Physical education & sport science university students	FL, TH	Compared with the control group, muscle directed static stretching for 12 weeks, showed increased ROM, decreased shear wave velocity of triceps surae, decreased passive torque and greater GM fascicle length. There were no significant changes in GL fascicle length and in GM and GL thickness
Blazevich et al. [24]	RCT	24	24	**-**	15	9	18.6 ± 0.9	18.6 ± 0.9	NR	FL	A 3-week stretching training increased dorsiflexion ROM and passive joint moment at end ROM in the stretched compared with the control group. Muscle and fascicle strain increased along with a decrease in muscle stiffness during stretch to a constant joint angle. Muscle length at end ROM increased without a change in fascicle length, fascicle rotation, tendon elongation and tendon stiffness, following training. No change in maximum voluntary contraction moment and rate of force development at any joint angle was observed
Brusco et al. [41]	CT	10	10	**-**	Unilateral design		24.4 ± 4.1		Untrained participants	TH	After 6 weeks of unilateral static stretching, hip ROM increased only in the experimental leg. Biceps femoris thickness was significantly inreased at all time points and semitendinous thickness and echo intensity significantly increased at 72 h post stretching. However, no significant differences were found between the stretched and the control leg
Freitas and Mil-Homens [23]	RCT	10	10	**-**	5	5	21.2 ± 0.8	21.2 ± 0.8	University students	FL, PA, TH	An 8-week stretching intervention significantly increased BF fascicle length and hip joint ROM in the stretching compared with the control group. No changes were found in BF muscle thickness and pennation angle
Kay et al. [21]	RCT	26	16	10	13	13	27.8 ± 8.0	27.8 ± 8.0	Recreationally active participants	FL, PA, TH	After a 6-week active stretch training program, significant increases were found in eccentric and isometric moments, stretch tolerance, elastic energy storage, VL thickness, pennation angle and tendon stiffness. No change was observed in VL resting fascicle length and VL passive muscle–tendon stiffness
Konrad and Tilp [26]	RCT	49	35	14	25	24	23.3 ± 3.1	22.9 ± 2.4	Police Cadets	FL, PA	Following 6 weeks of static stretching, ankle ROM increased in the intervention group compared with the control. However, GM fascicle length, pennation angle, muscle stiffness and tendon stiffness remained unaltered post-intervention
Lima et al. [43]	RCT	23	23	**-**	12	11	19.1 ± 1.4	9.0 ± 0.2	Physically active participants	FL, PA, TH	After 8 weeks of stretching no significant changes were observed in VL and BF muscle architecture, extension torque and knee flexion angle. However, knee extension angle increased significantly in the experimental compared with the control group
Longo et al. [16]	RCT	30	18	12	15	15	22.3 ± 0.8	23.4 ± 0.8	Recreationally active participants	FL, PA, TH	Compared to pre-intervention, a static stretching intervention of 12-weeks increased ankle ROM in the intervention group while muscle tendon complex stiffness decreased. No changes were found in triceps surae architecture (FL, PA, TH), and plantar flexors force generating capacity. No changes occurred in the control group in any variable
Mizuno [20]	CT	24	15	9	12	12	18.5 ± 0.7	18.8 ± 0.7	University students	PA, TH	A static stretching intervention of 8 weeks significantly increased ankle ROM and GM muscle thickness in the stretching compared with the control group. In addition, there were significant increases in plantar flexion one-repetition maximum strength and pennation angle in the stretching and the control group
Moltubakk et al. [17]	RCT	26	9	17	Unilateral design		22.0 ± 1.6		Recreationally active university students	FL, PA, THFL, PA, TH	Following 24 weeks of static stretching, ankle ROM increased and passive torque and normalized EMG amplitude at a standardized dorsiflexion decreased. Increases were seen in passive tendon elongation at a standardized force and in maximal passive muscle and tendon elongation. No changes were seen in tendon stiffness, resting tendon length or GM fascicle length. No changes were found in GM thickness and pennation angle in the stretched leg
Nakamura et al. [22]	RCT	40	40	**-**	14	13	21.4 ± 1.0	21.9 ± 1.3	University students	FL, PA, TH	High-intensity stretching improved ankle ROM and decreased muscle stiffness more than low intensity stretching. No significant changes were observed for muscle strength, drop jump height, and muscle architecture (FL, PA, TH) in both stretching groups compared to controls
					13		21.4 ± 1.1		University students		
Nakamura et al. [25]	RCT	18	18	**-**	9	9	21.1 ± 2.3	21.8 ± 0.8	NR	FL	Following 4 weeks of stretching, ankle ROM and gastrocnemius medialis MTJ displacement significantly increased while passive torque at 30° significantly decreased in the stretching compared with the control group. No increase was found in GM fascicle length in the stretching group
Panidi et al. [15]	RCT	21	**–**	21	Unilateral design		13.5 ± 1.4		Volleyball athletes	FL, PA, TH, ACSA	Following 12 weeks of stretching, ankle dorsiflexion increased in both legs with a greater increase in the stretched compared with the control leg. Fascicle length in the middle part of GM at rest and during stretching and fascicle length in the distal part of GL during stretching, increased only in the stretched leg. No changes were found in GM and GL penation angle and thickness. A greater increase was found in CSA and in one-leg jumping height in the stretched compared with the control leg
Peixinho et al. [37]	RCT	20	20	**–**	12	8	18.9 ± 0.5		Physically active	FL, PA, ACSA	Following 10 weeks of static stretching training maximum dorsiflexion, peak passive torque, and muscle–tendon unit maximum length significantly increased. No other differences were found related to muscle architecture
Şekir et al. [44]	CT	23	23	**-**	12	11	23.1 ± 3.1	22.2 ± 2.9	Recreational level athletes	FL, PA, TH	Following a 6-week stretching intervention, no significant increases were found for peroneal and tibial muscles architecture (FL, PA, TH)
Simpson et al. [14]	CT	22	11	11	Unilateral design		22.0 ± 2.0		NR	FL, PA, TH	After 6 weeks of overloaded static stretching gastrocnemius muscle thickness increased by 5.6%. Overall fascicles lengthened by 25% in the muscle tendon junction and 5.1% in the muscle belly. The fascicles in GL lengthened to a greater extent than in GM. Pennation angles remained unaltered in GM but decreased in GL. No change was observed in maximm voluntary contraction, voluntary activation, tendon length or thickness
Warneke et al. [45]	CT	27	16	11	Unilateral design		27.4 ± 3.1	26.8 ± 3.9	Athletically active subjects	TH	After a 6 weeks static stretching intervention using an orthosis, maximal isometric strength, 1RM and ROM significantly increased. In addition, there was a significant contralateral transfer in maximal strength. A significant increase was observed in muscle thickness in the GL of the stretched leg
Yahata et al. [18]	CT	16	16	**-**	Unilateral design		21.4 ± 1.5		NR	FL, PA, TH	After a 5 week stretching intervention, significant increases were found in maximum voluntary isometric contraction, at neutral ankle position. No changes were found in muscle architecture (FL, PA, TH) for both the intervention and the control legs
Participants total		467	342	125							

CT: controlled trial; RCT: randomized controlled trial; SG: stretching group; CG: control group; FL: fascicle length; PA: pennation angle; TH: muscle thickness; ACSA: anatomical cross sectional area; NR: not reported; GM: gastrocnemius medialis; GL: gastrocnemius lateralis; VL: vastus lateralis; BF: biceps femoris; ROM: range of motion; MTJ: muscle–tendon junction

Also, the following information was extracted from the included studies: (a) joints and muscles examined, (b) the stretching intervention characteristics (i.e., the duration of each stretching bout, the number of stretching exercises, the number of sets, and the frequency of stretching training per week). From these data, the following parameters were calculated: (a) daily stretching duration (duration of each stretching bout × number of sets × number of exercises), (b) the stretching duration per week (duration of daily stretching × number of stretching trainings per week) and (c) the total duration of the stretching intervention (stretching duration per week × number of weeks). Stretch intensity, expressed by the perceived rating of pain, was also extracted. The characteristics of the stretching protocols can be found in Table 2.

**Table 2:** Characteristics of the stretching interventions

Study	Stretching duration (bout) (s)	Number of exercises	Number of sets	Frequency per week	Daily stretch in a week (s)	Weekly load (s)	Study duration (weeks)	Total stretching duration	Stretching intensity
Akagi and Takahashi [42]	120	1	3	6	360	2160	5	10,800	Without suffering discomfort or pain
Andrade et al. [9]	45	2	5	5	450	2250	12	27,000	Max, onset of pain
Blazevich et al. [24]	30	1	8	7	240	1680	3	5400	Within the limit of pain
Brusco et al. [41]	60	1	8	2	480	960	6	5760	Max-tolerable
Freitas and Mil-Homens [23]	90	1	5	5	450	2250	8	18,000	POD
Kay et al. [21]	36	1	5	2	180	360	6	2160	POD
Konrad and Tilp [26]	30	1	4	5	120	600	6	3600	POD
Lima et al. [43]	30	1	3	3	90	270	8	2160	Preceding pain threshold
Longo et al. [16]	45	2	5	5	450	2250	12	27,000	POD
Mizuno [20]	30	1	4	3	120	360	8	2880	Without feeling pain
Moltubakk et al. [17]	60	4	4	7	240	1680	24	40,320	Without pain
Nakamura et al. [22]	60	1	3	3	180	540	4	2160	Between 6–7
	60	1	3	3	180	540	4	2160	Greatest tolerated dorsiflexion with no or little pain
Nakamura et al. [25]	60	1	2	7	120	840	4	3360	POD
Panidi et al. [15]	78.75	6	2	5	945	4725	12	56,700	POD
Peixinho et al. [37]	30	2	2	4	120	480	10	4800	Tolerable discomfort
Şekir et al. [44]	30	1	4	5	120	600	6	3600	Mild discomfort
	30	2	4	5	240	1200	6	7200	
Simpson et al. [14]	180	1	1	5	180	900	6	5400	Mild discomfort
Warneke et al. [45]	3600	1	1	7	3600	25,200	6	151,200	POD with an orthosis
Yahata et al. [18]	300	1	6	2	1800	3600	5	18,000	20% maximum voluntary contraction

POD: point of discomfort

### Data Synthesis and Meta-Analysis Methods

Data for the meta-analysis were obtained from all the included studies in the systematic review. Means and standard deviations for each variable of interest, before and after the intervention or control period, were extracted either from the Results section of the manuscript or from tables and figures. In the case of missing data, the corresponding authors of the included studies were contacted via email. Delta scores were calculated from the pre- and post-intervention means, by subtracting the baseline from the post-intervention values. Standard deviations for the delta scores were calculated using the following equation: $\sqrt {\left( {SD^{2} {\text{pre}} + SD^{2} {\text{post}}} \right){-}\left( {2 \times 0.70 \times {\text{SDpre}} \times {\text{SD post}}} \right){ }}$ [34]. The standardized mean difference approach, using the delta scores and SDs of the experimental and control groups, was then used. The meta-analysis was conducted by employing an inverse-variance, continuous, random-effects model, using the metafor package in R [38]. The syntax file can be found in the Additional file 9. Heterogeneity in the effects was determined by the Q and I^2^ statistic [36], using a cut off value of I^2^ = 75% as an index of considerable heterogeneity [35]. For each architectural characteristic (fascicle length, fascicle angle, and muscle thickness), an omnibus analysis was performed irrespective of the stretching protocol by AM. This was followed by separate analyses for each protocol and complemented by comparisons between high and low total stretching volume load as well as between high and low stretching intensity by AM. The cut-off value for the stretching volume load was determined according to the median split method (median = 5400 s) [39]. This median value represents the total stretching duration of 6 weeks of training performed five times per week, with each session including two stretching exercises of 30 s executed for three sets. Low-intensity studies included those which described stretch intensity as “no pain perception”, “stretching preceding pain threshold”, “pain between 6 and 7 on an analog scale ranging from 1 to 10”, and “without suffering discomfort” (Table 2). High-intensity studies included those which described pain perception as “highest or maximum tolerable”, “point of discomfort”, and “maximum tolerable after the onset of pain” (Table 2). Thus, primary outcomes were: (a) changes in fascicle length at rest and during stretching, (b) fascicle angle, and (c) muscle thickness. Subgroup analyses included differences according to stretching volume (high vs. low) and intensity (high vs. low). In addition, random effects meta-regression analyses were conducted using the total stretching volume load and stretching intensity as covariates (IBM SPSS Statistics Version 28.0, IBM Corporation, Armonk, New York, USA). Standardized mean differences (SMD) were characterized as trivial (< 0.2), small (0.2–0.6), moderate (0.6–1.2), large (1.2–2.0), very large (2.0–4.0), and extremely large (> 4.0) [40]. An alpha level of 0.05 was defined for the statistical significance of all the tests, apart from heterogeneity (*p* < 0.10). Moreover, Egger’s regression intercept test and visual inspection of the funnel plots were applied to detect possible publication bias.

## Results

### Results of the Search Procedure

**Fig. 1:**
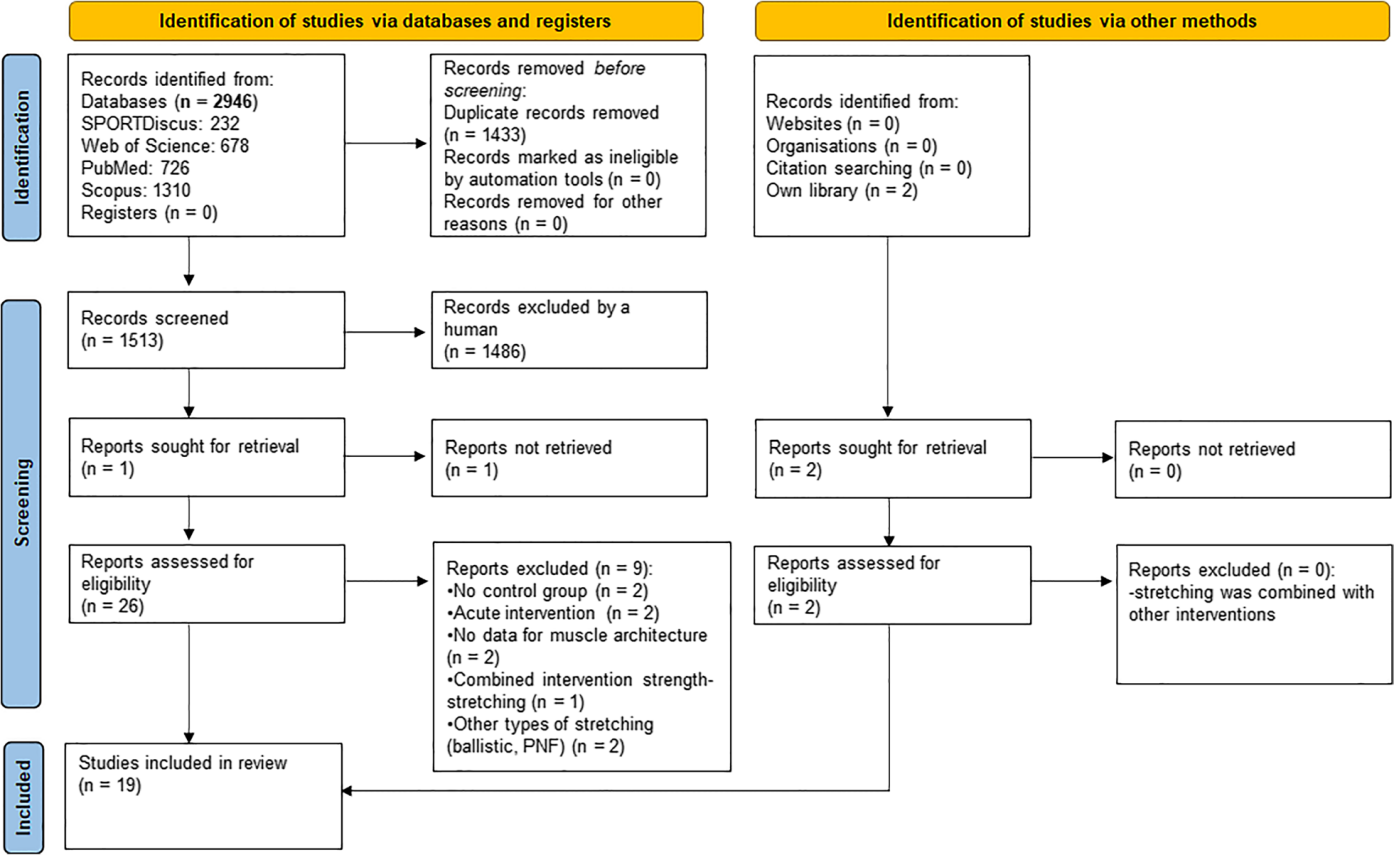
PRISMA flowchart illustrating different phases of the search and study selection

Initially, 2946 papers were retrieved. After duplicates were removed (n = 1433), 1513 papers remained for eligibility evaluation. Of these 1513 papers, 53 were reviews, 25 examined acute stretching interventions, 54 involved clinical populations, 122 involved animals, five were case reports, 15 conference papers, and 1212 were studies not directly relevant to the study purpose. Finally, 27 papers were eligible for this study, of which one paper could not be obtained, despite having contacted the corresponding author. The reference lists of the 26 remaining eligible studies were then checked for additional relevant studies. Following this additional search of the references and the inclusion of our own library, we identified and added two more relevant papers. After screening the full texts of the 28 eligible papers, 9 papers were excluded for different reasons (see Fig. 1). Therefore, 19 papers were finally included in this systematic review and were used in the meta-analyses. A flow chart of the search process is presented in Fig. 1.

### Characteristics of the Included Studies

The 19 eligible studies were published between 2013 and 2022 and included 467 participants (342 males), aged 21.1 ± 1.6 years. All the eligible studies used static stretching and all the protocols targeted the lower limbs. Their characteristics are presented in Table 1. Out of the 19 eligible studies, five were CTs [14, 18, 20, 37, 41] and 14 were RCTs [9, 15–17, 21–26, 42–45]. Fourteen studies examined resting fascicle length (30 entries), six studies examined fascicle length during stretching (9 entries), 15 studies (31 entries) examined muscle thickness and 11 studies (25 entries) examined fascicle angle. A detailed description of the stretching protocols (i.e., the duration of each stretching bout, number of exercises and sets, joints involved, and total stretching duration) is provided in Table 2.

### Risk of Bias Assessment

**Fig. 2:**
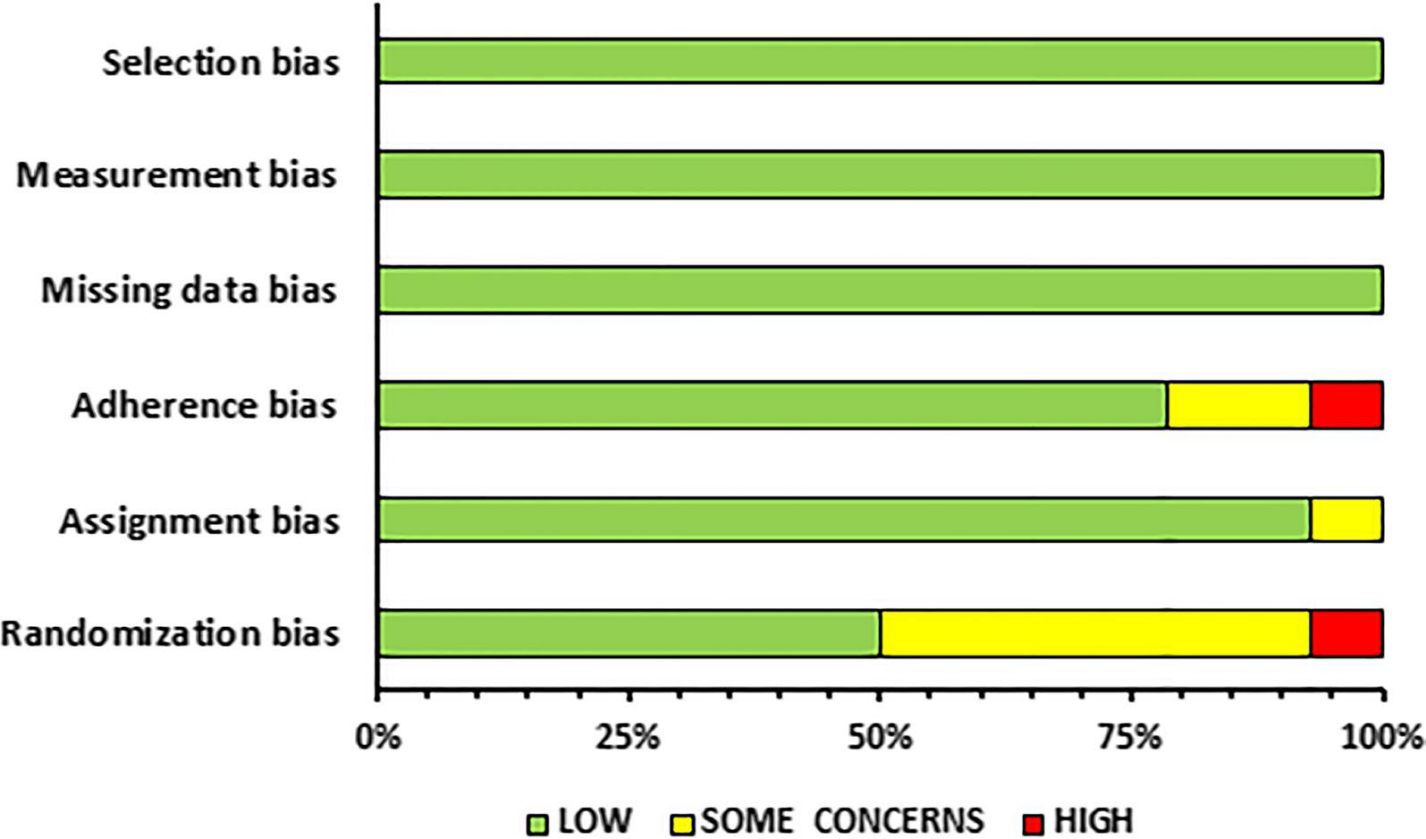
Summary of risk of bias assessment for randomized controlled trials

**Fig. 3:**
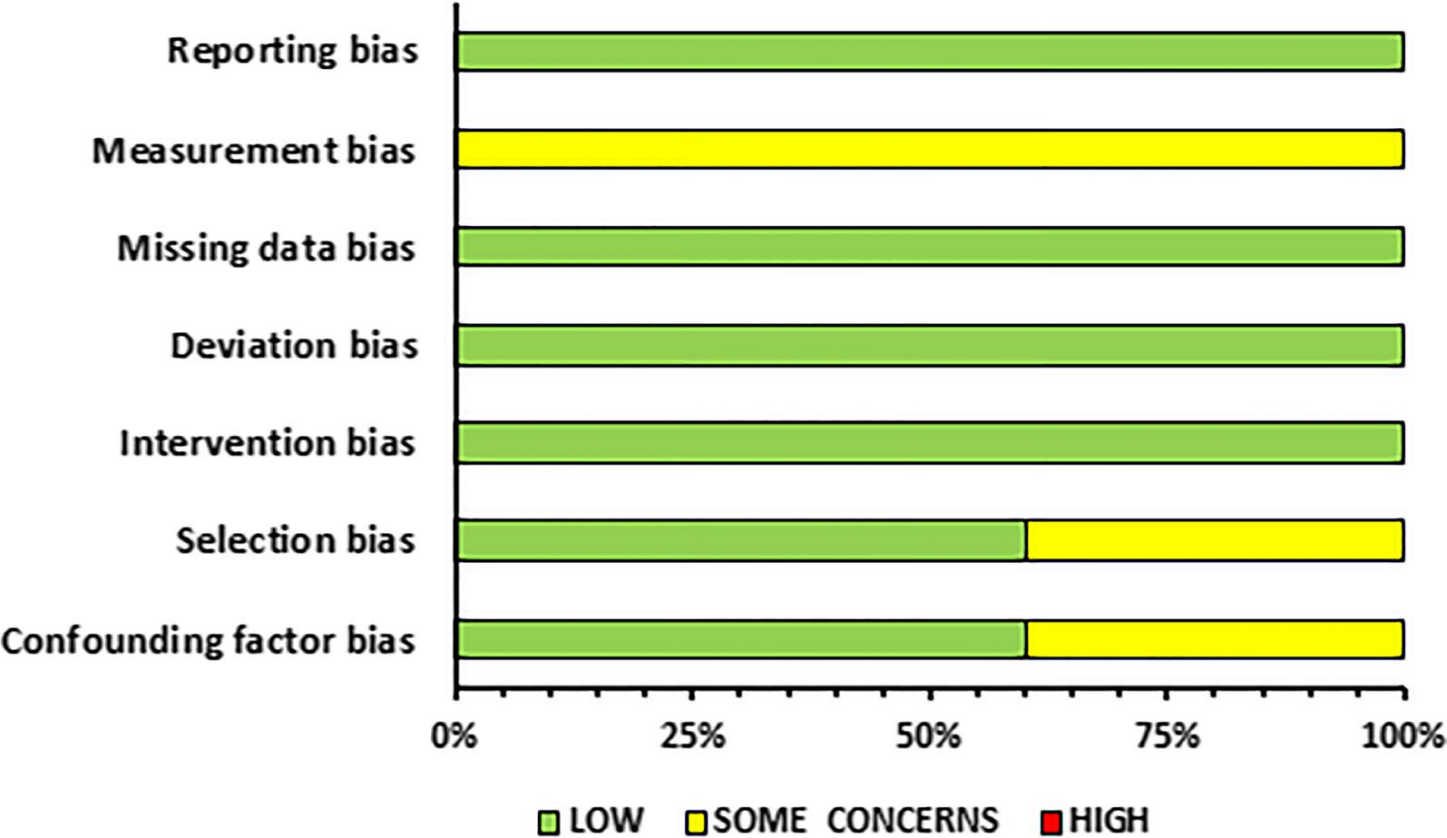
Summary of risk of bias assessment for controlled trials

A summary of the risk of bias assessment is provided in Figs. 2 and 3 for the RCTs and CTs, respectively. Detailed descriptions of the risk of bias assessment for all the included studies are presented in the Additional files 3 and 4: S3 and S4 for the RCTs and CTs, respectively.

### Primary Outcomes

**Fig. 4:**
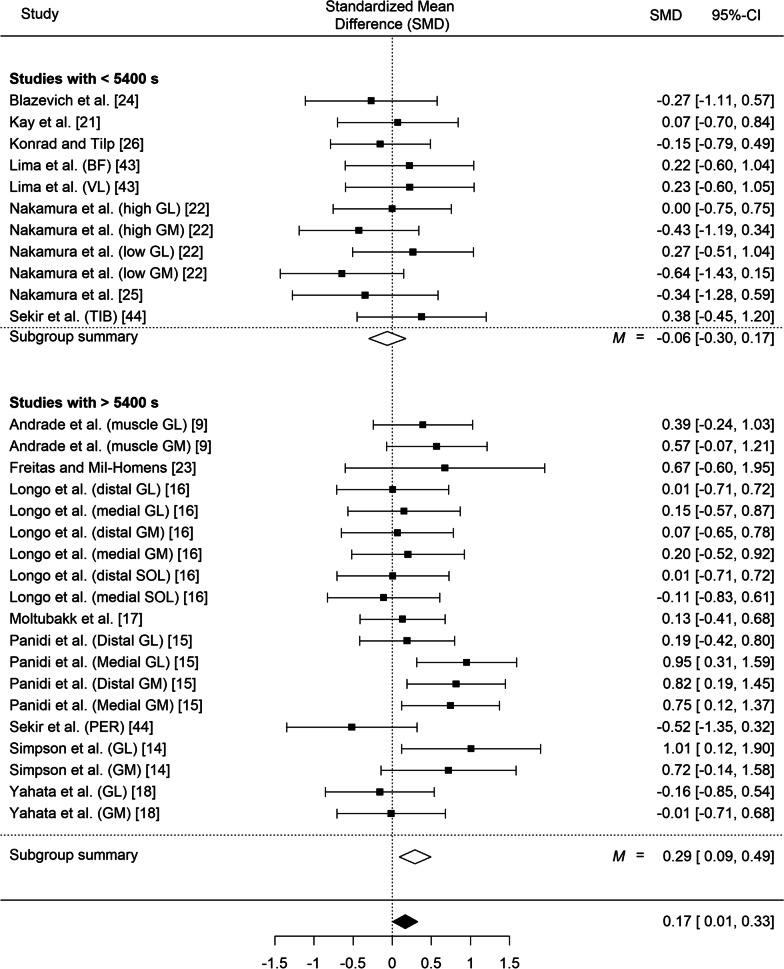
Effect of static stretching training on fascicle length at rest (overall effect and according to the total stretching volume). 95% CI: Confidence Interval. *Note*: GM: gastrocnemius medialis; GL: gastrocnemius lateralis; VL: vastus lateralis; BF: biceps femoris; SOL: soleus; PER: peroneus muscle; TIB: tibialis muscle

**Fig. 5:**
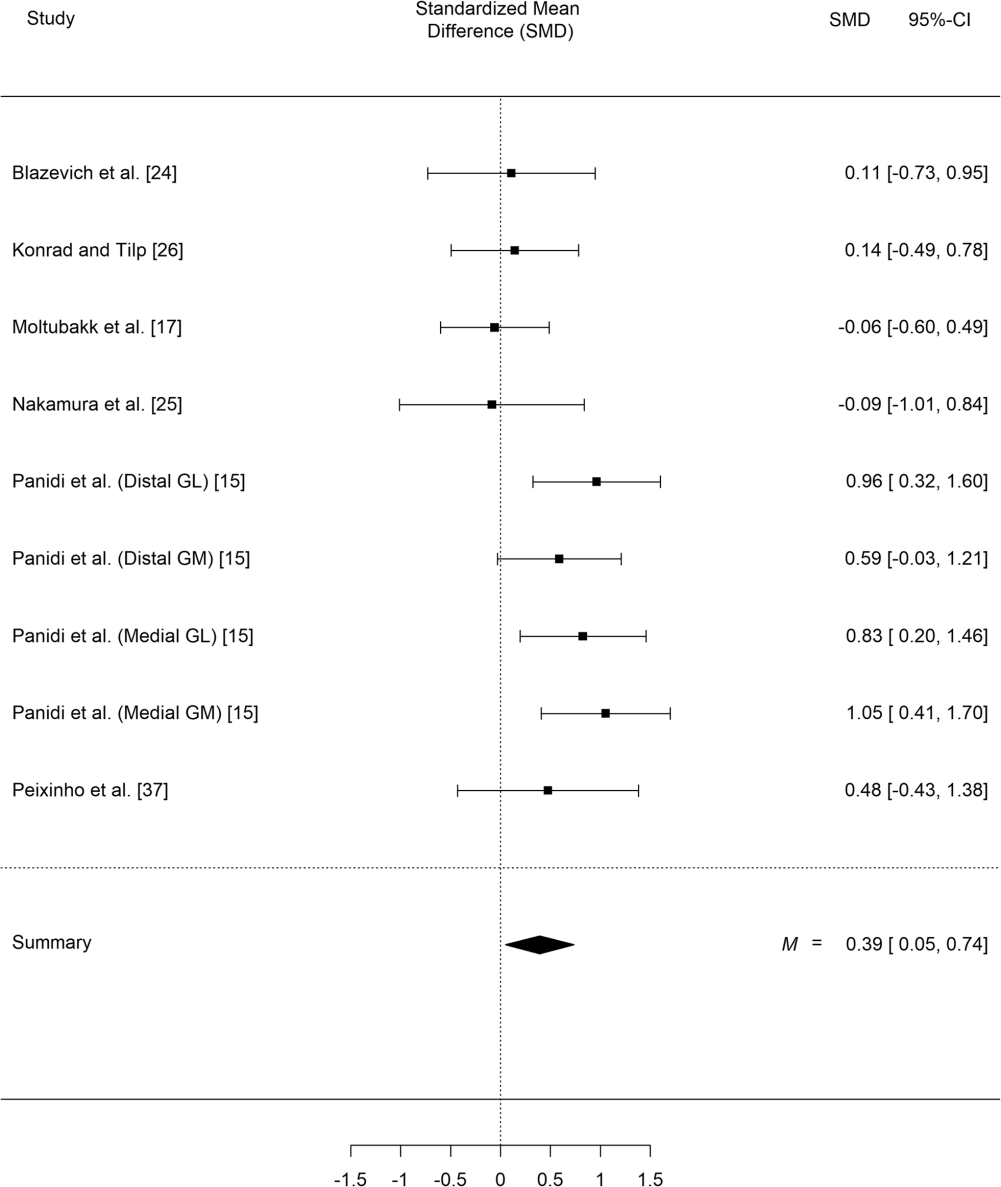
Effect of static stretching training on fascicle length during stretching. 95% CI: Confidence Interval. *Note*: GM: gastrocnemius medialis; GL: gastrocnemius lateralis

Our meta-analysis of fascicle length, which included all the studies, regardless of their intervention protocol but in which we took into account the nesting structure of some data (given that in some studies the same muscle was assessed twice at two different muscle parts) indicated statistically significant differences in resting fascicle length between the experimental groups and the control groups (SMD = 0.17; *SE* = 0.08, *z* = 2.03, *p* = 0.042, 95% CI 0.01 to 0.33; *Q*(29) = 35.56, *p* = 0.19, I^2^ = 24.15%; Fig. 4). Likewise, stretching training yielded significant differences in fascicle length during stretching (SMD = 0.39; *SE* = 0.18, *z* = 2.23, *p* = 0.026, 95% CI 0.05 to 0.74; *Q*(8) = 13.49, *p* = 0.10, I^2^ = 46.90%; Fig. 5).

**Fig. 6:**
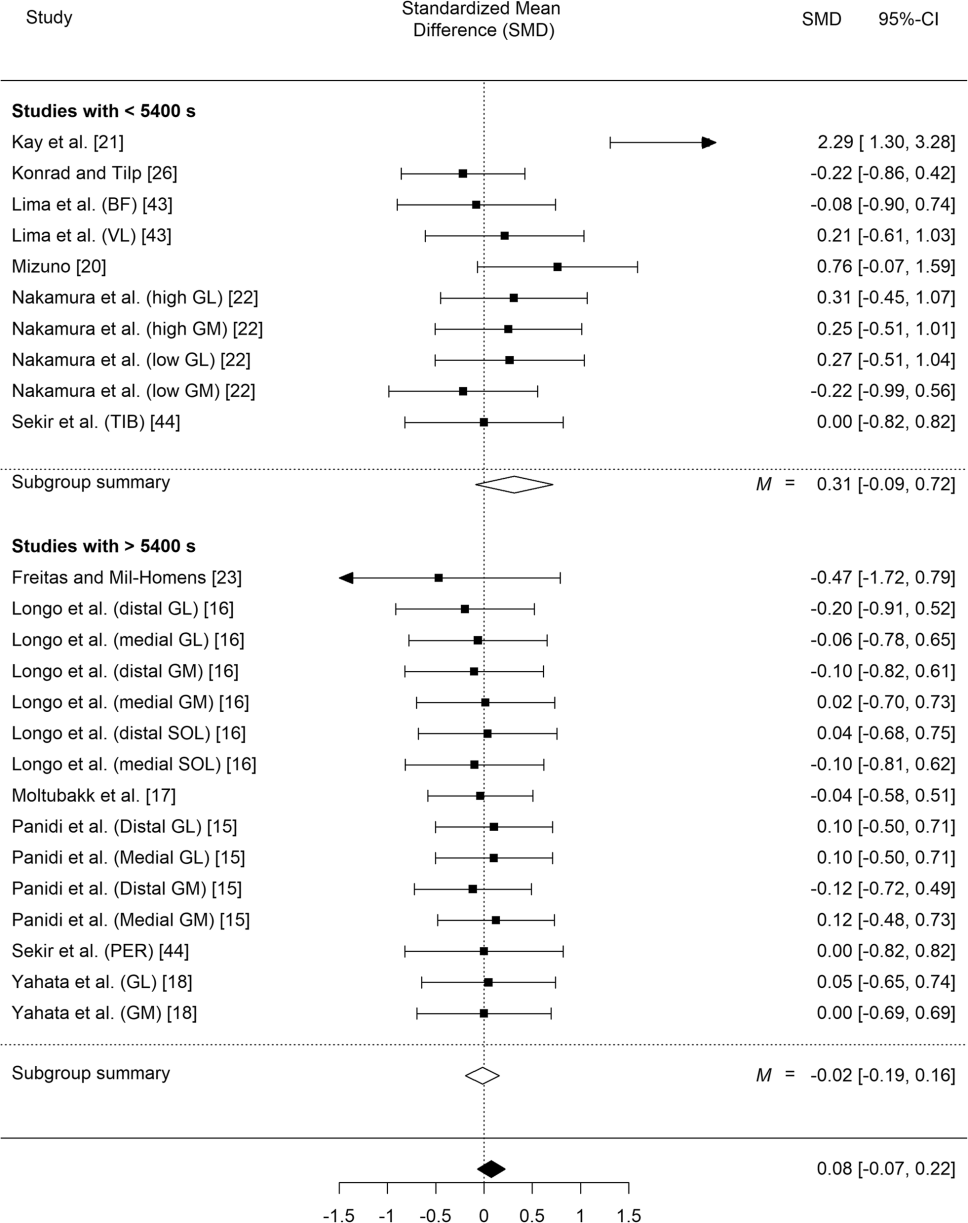
Effect of static stretching training on fascicle angle (overall effect and subgroups comparisons by total stretching volume). 95% CI: Confidence Interval. *Note*: GM: gastrocnemius medialis; GL: gastrocnemius lateralis; VL: vastus lateralis; BF: biceps femoris; SOL: soleus; PER: peroneus muscle; TIB: tibialis muscle

No differences were found either in fascicle angles (SMD = 0.08, *SE* = 0.07, *z* = 1.03, *p* = 0.30; 95% CI − 0.07 to 0.22; *Q*(24) = 26.97, *p* = 0.31, I^2^ = 0.00%; Fig. 6) or in muscle thickness following the stretching interventions (SMD = 0.11; *SE* = 008, *z* = 1.35, *p* = 0.18; 95% CI − 0.05 to 0.28; *Q*(30) = 45.99, *p* = 0.03; I^2^ = 33.22%).

### Subgroup and Meta-Regression Analyses

Although we found no systematic heterogeneity in our analyses, we further examined the degree to which the research protocol (i.e., less vs. more than 5400 s) would reveal differences between the experimental and control groups, given that the *Q* statistic and its derivative, *I*^2^, are insensitive to detect heterogeneity when meta-analyzing a small number of studies [46].

#### Fascicle Length by Stretching Volume Interaction

Out of the 30 entries analyzed, 11 had a low total volume (i.e., < 5400 s) and 19 had a high total volume (≥ 5400 s). The low and high-volume load groups differed in total stretching volume (3030 ± 1057 vs. 24,953 ± 17,099 s, *p* = 0.003), due to the 2.5-fold longer stretching bout duration 104 ± 92 vs. 42 ± 15 s) and the longer intervention duration in the high vs. low volume load group (10.6 ± 6.2 vs. 5.1 ± 1.6 weeks, respectively, *p* = 0.028), while the number of exercises, sets and the frequency of training per week were similar. Interestingly, whereas no differences were found among the (*n* = 11) studies which induced a low total volume (i.e., < 5400 s), *SMD* = − 0.06; *SE* = 0.12, *z* = − 0.52, *p* = 0.60. 95% CI − 0.30 to 0.17; *Q*(10) = 6.46, *p* = 0.78; *I*^2^ = 0.00%, such differences emerged among the (*n* = 19) studies which induced a high total volume (i.e., > 5400 s) *SMD* = 0.29; *SE* = 0.10, *z* = 2.85, *p* = 0.004, 95% CI 0.09 to 0.49; *Q*(18) = 22.79, *p* = 0.20; *I*^2^ = 26.68%. A comparison of the standardized means of the two groups showed statistically significant differences (*z* = − 2.25, *p* = 0.024). Random effects meta-regression analysis also showed that total stretching volume is a moderator of longitudinal fascicle increases (*p* = 0.02, R^2^ = 0.76).

#### Fascicle Length by Stretching Intensity Interaction

**Fig. 7:**
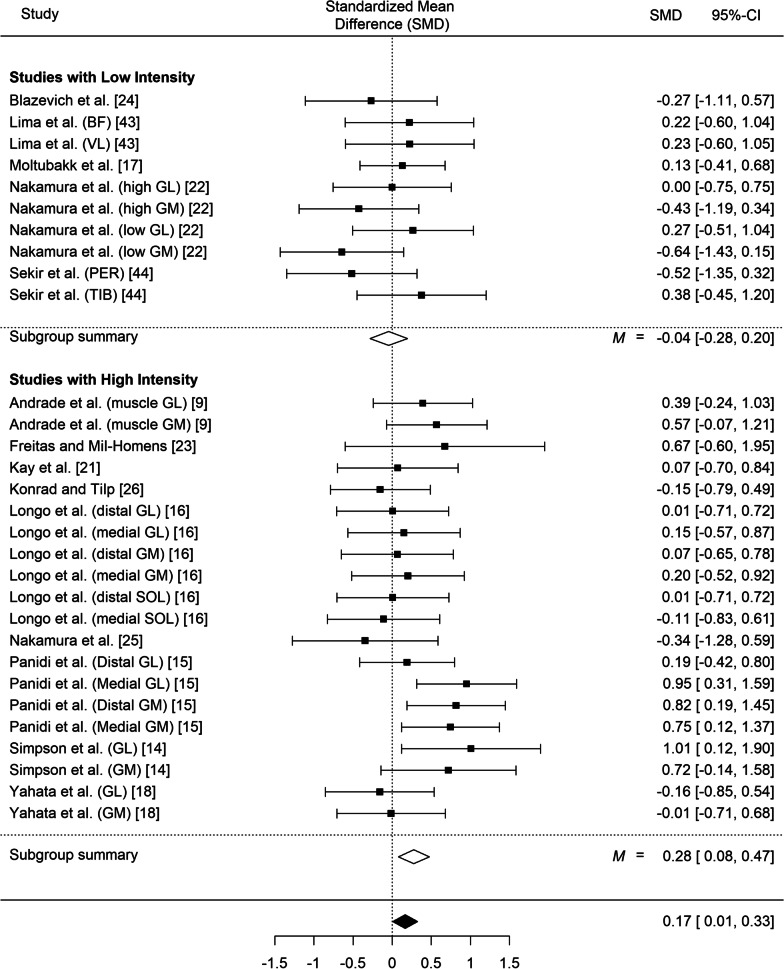
Effect of high and low stretching intensity on fascicle length; 95% CI: Confidence Interval. *Note*: GM: gastrocnemius medialis; GL: gastrocnemius lateralis; VL: vastus lateralis; BF: biceps femoris; SOL: soleus; PER: peroneus muscle; TIB: tibialis muscle

Out of the 30 entries analyzed, 9 had low intensity and 19 had high intensity. Only the high stretching intensities induced small increases in fascicle length following stretching (SMD = 0.28, *SE* = 0.10, z = 2.77, *p* = 0.006; 95% CI 0.08 to 0.47; *Q*(19) = 22.84, *p* = 0.24; *I*^2^ = 24.83%; Fig. 7). In contrast, low stretching intensities did not affect fascicle length in the experimental groups (SMD = − 0.05, *SE* = 0.12, *z* = − 0.36; *p* = 0.72; 95% CI − 0.28 to 0.20; *Q*(9) = 7.53, *p* = 0.58; *I*^2^ = 0%; Fig. 7). A comparison of the two models showed statistically significant differences (*z* = − 2.04, *p* = 0.042). Random effects meta-regression analysis showed that stretching intensity is a moderator of longitudinal fascicle increases (*p* < 0.04, R^2^ = 0.52).

#### Fascicle Angle by Stretching Volume Interaction

Out of the 25 entries analyzed, 10 had low volume and 15 had high volume. High or low stretching volumes did not induce changes in fascicle angle following stretching (SMD = − 0.02, *SE* = 0.09, *z* = − 0.19, *p* = 0.86; 95% CI − 0.19 to 0.16,; *Q*(14) = 1.55, *p* = 1.00, *I*^2^ = 0.00% and SMD = 0.32, *SE* = 0.21, *z* = 1.53, *p* = 0.13; 95% CI − 0.09 to 0.72, *Q*(9) = 22.31; *I*^2^ = 61.27%, respectively; Subgroup difference: z = 1.45, *p* = 0.14; Fig. 6).

#### Fascicle Angle by Stretching Intensity Interaction

Out of the 25 entries analyzed, 11 had low intensity and 14 had high intensity. High or low stretching intensities did not induce changes in fascicle angle following stretching, (*SMD* = 0.15; *SE* = 0.18, *z* = 0.84, *p* = 0.40, 95% CI − 0.20 to 0.50; *Q*[13] = 21.67, *p* = 0.06; *I*^2^ = 60.01% and SMD = 0.12, *SE* = 0.12, *z* = 1.00, *p* = 0.32; 95% CI − 0.11 to 0.35, *Q*[13] = 4.38, *p* = 0.93; *I*^2^ = 0.00%, respectively; Subgroup difference: z = − 0.16, *p* = 0.88).

#### Muscle Thickness by Stretching Volume Interaction

Out of the 31 entries analyzed, 11 had low volume and 20 had high volume. High or low stretching volumes did not induce changes in muscle thickness following stretching, (SMD = 0.11, *SE* = 0.10, *z* = 1.16, *p* = 0.25; 95% CI − 0.08 to 0.30; *Q*(19) = 25.06, *p* = 0.16; I^2^ = 29.33% and SMD = 0.13, *SE* = 0.18, *z* = 0.76, *p* = 0.45; 95% CI − 0.21 to 0.48; *Q*(10) = 20.89, *p* = 0.022; *I*^2^ = 51.64%, respectively; subgroup difference: *z* = 011, *p* = 0.92).

#### Muscle Thickness by Stretching Intensity Interaction

**Fig. 8:**
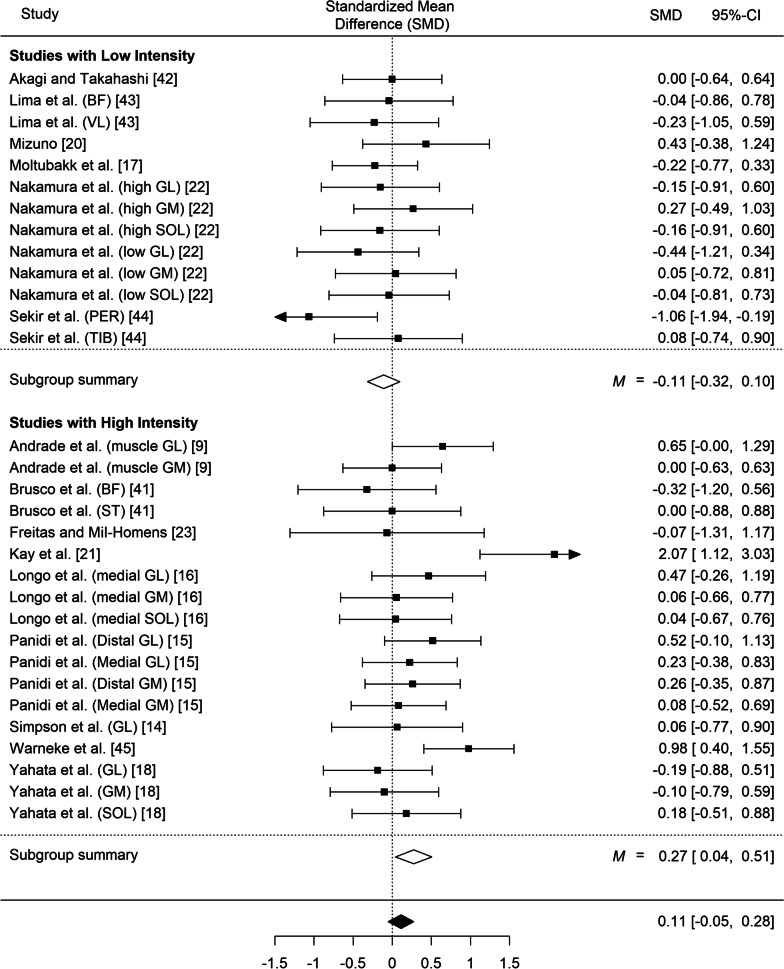
Effect of static stretching training on muscle thickness (overall effect and subgroups comparisons by stretching intensity). 95% CI: Confidence Interval. *Note*: GM: gastrocnemius medialis; GL: gastrocnemius lateralis; VL: vastus lateralis; BF: biceps femoris; SOL: soleus; PER: peroneus muscle; TIB: tibialis muscle; ST: semitendinosus

Out of the 31 entries analyzed, 13 had low intensity and 18 had high intensity. Subgroup analysis showed that stretching training with high intensity induced a small increase in muscle thickness, (SMD = 0.27, *SE* = 0.12, *z* = 2.31, *p* = 0.021; 95% CI 0.04 to 0.51, *Q*(17) = 29.04, *p* = 0.034; *I*^2^ = 42.49%), while low intensity stretching had no effect (SMD = − 0.11, *SE* = 0.11, *z* = − 1.03, *p* = 0.30; 95% CI − 0.32 to 0.10, *Q*(12) = 8.72, *p* = 0.73; *I*^2^ = 0%; subgroup difference: *z* = − 2.41, *p* = 0.016, Fig. 8).

### Confidence in Cumulative Evidence

Detailed GRADE analyses can be found in the Additional file 5: S5. In this study, 14 randomized controlled trials and five controlled trials were included thus, GRADE started assuming high quality. The quality of evidence was not downgraded for Risk of Bias, inconsistency of the results or indirectness but was downgraded by one level for publication bias. According to GRADE guidelines, we used the threshold of 800 participants as a cut off point for imprecision on the results of the pooled analysis. Thus, fascicle length during stretching (n = 319) and fascicle angle analyses (n = 760) were downgraded by one level. Since a dose–response effect was found for fascicle length, the studies examining longitudinal fascicle length were upgraded. For the same reason, studies examining the effects of stretching intensity on muscle thickness were also upgraded. Overall, the analysis showed that we can have considerable confidence that the true effect is similar to the estimated effect. Visual inspection of the funnel plots implied no publication bias (see Additional files 6, 7, 8: Figs. 1–3 for funnel plots). In addition, Egger’s regression intercept test revealed no publication bias for fascicle length, fascicle angle and muscle thickness (intercept = 0.525, *p* = 0.313, − 0.743, *p* = 0.292 and − 0.195, *p* = 0.802, respectively).

## Discussion

The aim of this systematic review and meta-analysis was to examine the effects of static stretching training on muscle architecture. The main meta-analysis, including a total of 19 studies and 467 participants, indicated that static stretching training induces trivial increases in fascicle length at rest and small increases in fascicle length during stretching in healthy participants. As shown by subgroup analyses and meta-regression, increases in fascicle length and muscle thickness are moderated by stretching volume and intensity. Specifically, high stretching volumes and intensities induce longitudinal fascicle growth, while high stretching intensities result in increased muscle thickness. Fascicle angle remains unaffected by static stretching training.

It has been shown that fascicle length reflects the number of sarcomeres in series and is related to maximum muscle excursion [29]. In animal studies, long-term immobilization in a lengthened position induces increases in muscle fiber length [7, 47, 48], possibly due to the addition of sarcomeres in series [8]. However, an increase in fascicle length following stretching in humans has not been clearly demonstrated to date. The main meta-analysis showed a significant increase in resting fascicle length following static stretching training which approached a small magnitude change (SMD = 0.17, *p* = 0.042). Since static stretching is commonly used in sports, rehabilitation, and clinical settings [9], even trivial changes in fascicle length may be of importance.

During stretching, mechanical stress and, most importantly, total time under tension, contribute to morphological adaptations [49]. It has been hypothesized that when a muscle is systematically stretched to long muscle lengths, sarcomere number in series may increase to reduce passive tension and to maintain optimal actin-myosin overlap [49, 50]. The results of the present meta-analysis indicated that only high stretching volumes or high stretching intensities induce increases in fascicle length (SMD = 0.29, *p* = 0.004 and SMD = 0.28 *p* = 0.006, respectively) while low stretching volumes and intensities did not induce changes in muscle morphology (SMD = − 0.06, *p* = 0.60 and SMD = − 0.04 *p* = 0.72, respectively). Thus, it seems that total mechanical stress, as expressed by volume load and intensity, is an important modulator of the increases in fascicle length during stretching training [11, 17]. For example, significant increases in gastrocnemius medialis fascicle length at rest and in gastrocnemius lateralis fascicle length during stretching, were found after 12 weeks of daily high-intensity and high volume stretching [9, 15]. In contrast, a 6-month intervention using low intensity stretching did not result in fascicle length changes of gastrocnemius [17].

The cut-off value for the stretching volume in the present study (i.e., 5400 s or 90 min), represents the total stretching duration of six 30 s sets performed five times per week for 6 weeks, and is higher than what is commonly used in sports practice [51]. The high and low volume subgroups differed largely in total stretching volume (3030 ± 1057 vs. 24,953 ± 17,099 s, *p* = 0.003), due to the 2.5-fold longer stretching bout duration and the longer intervention duration in the high vs. low volume load group, while the number of exercises, sets and the frequency of training per week were similar (Table 2). These findings highlight the importance of long stretching bout duration (from 30 to 300 s, average of 101 s) to achieve an increase in fascicle length. Notably, these stretching bout durations are much higher than those used by athletes (10–20 s, average of 14.5 s) during their practice [51], suggesting that longer stretching bouts should be employed when morphological changes in muscles are required. Since prolonged stretching duration (> 60-s per muscle group per session) may acutely impair strength and power parameters [52, 53] it is suggested that long duration and high intensity stretching bouts should be included in a separate flexibility training session. Additionally, the difference in the intervention duration between high and low volume groups (10.6 ± 6.2 vs. 5.1 ± 1.6 weeks, respectively, *p* = 0.028) may suggest that, besides stretching bout duration, morphological adaptations may require longer time to occur. Although some fascicle length increases were reported following 6 weeks of overloaded stretch training [14], the greater fascicle length that is observed in cross-sectional studies in dancers [17] and gymnasts [54, 55] compared with athletes from other sports, suggests that long-term stretching training with high-volume and intensity is important for adaptations in muscle morphology. In this respect, more evidence is needed regarding the effects of long-term stretching protocols on longitudinal fascicle growth, applied throughout childhood and adolescence, which may be a suggestion for future studies.

A greater fascicle length during stretching was observed in the experimental groups, compared with the control groups, with a small effect size (SMD = 0.39, *p* = 0.026). Previous cross-sectional studies observed greater fascicle length during stretching in flexibility trained compared to untrained adults [24, 56] and the same was found in flexibility trained children [54]. The limited evidence provided by the few studies that measured fascicle length during stretching (n = 6), has shown relatively larger increases compared with those observed at rest (10.9 vs.5.3%) [15, 24]. The large increases in fascicle extensibility found in this meta-analysis are an important finding. It is not known if the increased fascicle extensibility following stretching training reflects changes in series elastic (e.g., the muscle internal aponeuroses, the structural protein “titin”, the elastic elements in the cross-bridges aponeurosis) or contractile elements (i.e. sarcomeres), and it remains undetermined how these changes may affect the mechanics of muscle contraction, the metabolic cost of movement and the storage and release of elastic energy [57].

The main meta-analysis showed no differences in fascicle angle following static stretching training (SMD = 0.08, *p* = 0.30) and no changes were found following high or low stretching volumes (*p* = 0.86 and *p* = 0.13, respectively) and intensities (*p* = 0.40 and *p* = 0.32, respectively). In line with the results of this systematic review, several studies reported unaltered fascicle angles following stretching training [16, 26], while one study reported trivial decreases in gastrocnemius lateralis fascicle angle [14]. Fascicle angle, defined as the angle between a fascicle’s orientation and the aponeurosis axis, is thought to determine force contribution of the fascicle during skeletal movement [58]. However, it has recently been suggested that fascicle angle represents predominantly a “packing” strategy with little functional significance and unrelated to the magnitude of force generation through the tendon structure [58]. In this respect, current evidence suggests that the tension generated by stretching induces no changes in fascicle angle.

Also, this meta-analysis showed that there was no difference in muscle thickness following static stretching training (SMD = 0.11, *p* = 0.18). Most studies reported no changes in muscle thickness following static stretching training (Fig. 8). However, subgroup analyses showed a small effect of high intensity stretching on muscle thickness (SMD = 0.27, *p* = 0.021, subgroup difference *p* = 0.016). As can be seen in Fig. 8, this was due to four studies that combined high intensity and very high total volume protocols (i.e., accumulation of > 450 min of total stretching duration) applied to the gastrocnemius muscle [9, 15, 16, 37]. Notably, the fifth study which showed a large improvement in muscle thickness with high-intensity, but low-volume stretching, involved the vastus lateralis muscle [21]. Thus, it may be argued that a combination of high intensity and very high volume of stretching (> 7.5 h) is required to increase muscle thickness of the gastrocnemius [9, 15, 16, 37]. Despite the apparent importance of high intensity and high-volume combination to induce a hypertrophic response following static stretching training, further investigation is required to determine the magnitude and the characteristics or the appropriate programs.

Regarding muscle cross-sectional area, only two studies examined [15, 37] the effect of static stretching training on gastrocnemius muscle anatomical cross-sectional area in humans. In one study examining adolescent female volleyball players it was found that intense static stretching increased cross-sectional area in the gastrocnemius of the stretched leg (by 23%), while the non-stretched leg also hypertrophied, albeit by a significantly smaller percentage (13%, *p* < 0.01) [15]. The difference in the percent increase of the cross-sectional area between the stretched and the control legs may be attributed to the interaction of volleyball and stretching training, which further enhanced muscle hypertrophy [15]. In the second study that measured the effects of stretching on cross-sectional area, no changes were found in the gastrocnemius muscle following 10-weeks of low volume and intensity stretching [37].

Since high volume and high intensity static stretching has the potential to induce longitudinal fascicle growth, muscle thickness and muscle cross sectional area, future studies should examine how these changes in muscle morphology may influence muscle mechanical function (e.g., force–length relationship). Some interventions indicate that increased fascicle length may shift the optimal muscle length for force production [2] and may widen the entire force–length relationship [59], but this remains to be verified for stretching training interventions. Since some injuries occur close to the end of the range of motion with the muscle in a lengthened state, this shift of the force–length relationship could play a role in reducing such injuries [52]. In addition, future research should examine the effect of longitudinal fascicle growth following stretching on velocity of contraction during shortening [49], as well as on the torque–angle relationship.

## Limitations

One limitation is that in this systematic review the effects of stretching training could not be separated for males and females, as only one study reported results for females [15], while seven out of the 19 studies reported collective values for both sexes [9, 16, 17, 20, 21, 26, 45]. Furthermore, comparisons between athletic and non-athletic populations were not feasible, as only one study included an athletic population [15]. Another limitation concerns the characterization of stretching intensity, which was based on perceived discomfort and pain and not on any objective measures of intensity. This is an inherent limitation of almost all stretching interventions which should be addressed in future studies. Finally, most of the included studies examined the ankle joint (15 out of 19 studies), and there was limited information regarding other joints.

## Conclusions

Static stretching training induces trivial increases in fascicle length at rest and small increases in fascicle length during stretching in young, healthy participants. High volumes of static stretching and high stretching intensities are necessary to induce increases in fascicle length and muscle thickness, while fascicle angle remains unaffected by static stretching. These results show that long-term static stretching, using extended bouts of intense muscle elongation, may modify muscle architecture, with possible effects on muscle function. In that respect, static stretching may be used not only to increase ROM, but also to enhance muscle performance, either alone or in combination with other interventions, in health and disease.

## Supplementary Information

**Additional file 1**. Preferred Reporting Items for Systematic Reviews and Meta-Analyses (PRISMA) checklist.

**Additional file 2**. Search algorithm in PubMed, SCOPUS, Web of Science, and SPORTDiscus.

**Additional file 3**. Risk of Bias assessment for Randomized Controlled Trials.

**Additional file 4**. Risk of Bias assessment for Controlled Trials.

**Additional file 5**. GRADE analysis.

**Additional file 6: Fig. 1**. Funnel plot for fascicle length.

**Additional file 7: Fig. 2**. Funnel plot for fascicle angle.

**Additional file 8: Fig. 3**. Funnel plot for muscle thickness.

**Additional file 9**. Syntax file for R.

## Data Availability

Data are available at https://figshare.com/filename:10.6084/m9.figshare.20364738.

## Abbreviations

CI: Confidence interval
CTs: Controlled trials
GRADE: Grading of Recommendations, Assessment, Development and Evaluations
PICOS: Population, Intervention, Comparison, Outcome, Study Design
PRISMA: Preferred Reporting Items for Systematic reviews and Meta-Analyses
PROSPERO: International Prospective Register of Systematic Reviews
RCTs: Randomized controlled trials
RoB: Risk of Bias
ROBINS-I: Risk of Bias in Non-randomized Studies-of Interventions
ROM: Range of motion
SMD: Standardized Mean Difference
