# Supplementary material for: Muscle Architecture Adaptations to Static Stretching Training: A Systematic Review with Meta-Analysis
Source: Sports Med Open. 2023 Jun 15;9:47. doi: 10.1186/s40798-023-00591-7 (PMC10271914; doi:10.1186/s40798-023-00591-7)
Supplement: Supplementary file 2 — Additional file 2. Search algorithm in PubMed, SCOPUS, Web of Science, and SPORTDiscus. [file 40798_2023_591_MOESM2_ESM.docx]

**Supplementary file 2 (S2)**

**Search algorithm in PubMed**

("stretch training"[Title/Abstract] OR "stretching training"[Title/Abstract] OR "stretch intervention*"[Title/Abstract] OR "stretch program*"[Title/Abstract] OR "stretching program*"[Title/Abstract] OR "static stretch*"[Title/Abstract] OR "stretch*"[Title/Abstract] OR "flexibility training"[Title/Abstract] OR "flexibility intervention*"[Title/Abstract] OR "flexibility program*"[Title/Abstract]) AND ("architect*"[Title/Abstract] OR "fascicle"[Title/Abstract] OR "pennation"[Title/Abstract] OR "thickness"[Title/Abstract] OR "cross-sectional area"[Title/Abstract]) AND "muscle"[Title/Abstract])

**Search algorithm in Scopus**

("stretch training" OR "stretching training" OR "stretch intervention*" OR "stretch program*" OR "stretching program*" OR "static stretch*" OR "stretch*" OR "flexibility training" OR "flexibility intervention*" OR "flexibility program*") AND ("architect*" OR "fascicle" OR "pennation" OR "thickness" OR "cross-sectional area") AND "muscle"

**Search algorithm in Web of Science**

((TI=(("stretch training" OR "stretching training" OR "stretch intervention*" OR "stretch program*" OR "stretching program*" OR "static stretch*" OR "stretch*" OR "flexibility training" OR "flexibility intervention*" OR "flexibility program*") AND ("architect*" OR "fascicle" OR "pennation" OR "thickness" OR "cross-sectional area") AND "muscle")) OR AB=(("stretch training" OR "stretching training" OR "stretch intervention*" OR "stretch program*" OR "stretching program*" OR "static stretch*" OR "stretch*" OR "flexibility training" OR "flexibility intervention*" OR "flexibility program*") AND ("architect*" OR "fascicle" OR "pennation" OR "thickness" OR "cross-sectional area") AND "muscle")) OR KP=(("stretch training" OR "stretching training" OR "stretch intervention*" OR "stretch program*" OR "stretching program*" OR "static stretch*" OR "stretch*" OR "flexibility training" OR "flexibility intervention*" OR "flexibility program*") AND ("architect*" OR "fascicle" OR "pennation" OR "thickness" OR "cross-sectional area") AND "muscle")

**Search algorithm in SPORTDiscus**

TI ( ("stretch training" OR "stretching training" OR "stretch intervention*" OR "stretch program*" OR "stretching program*" OR "static stretch*" OR "stretch*" OR "flexibility training" OR "flexibility intervention*" OR "flexibility program*") AND ("architect*" OR "fascicle" OR "pennation" OR "thickness" OR "cross-sectional area") AND "muscle" ) OR AB ( ("stretch training" OR "stretching training" OR "stretch intervention*" OR "stretch program*" OR "stretching program*" OR "static stretch*" OR "stretch*" OR "flexibility training" OR "flexibility intervention*" OR "flexibility program*") AND ("architect*" OR "fascicle" OR "pennation" OR "thickness" OR "cross-sectional area") AND "muscle" ) OR KW ( ("stretch training" OR "stretching training" OR "stretch intervention*" OR "stretch program*" OR "stretching program*" OR "static stretch*" OR "stretch*" OR "flexibility training" OR "flexibility intervention*" OR "flexibility program*") AND ("architect*" OR "fascicle" OR "pennation" OR "thickness" OR "cross-sectional area") AND "muscle" )
