# Supplementary material for: Muscle Architecture Adaptations to Static Stretching Training: A Systematic Review with Meta-Analysis
Source: Sports Med Open. 2023 Jun 15;9:47. doi: 10.1186/s40798-023-00591-7 (PMC10271914; doi:10.1186/s40798-023-00591-7)
Supplement: Supplementary file 3 — Additional file 3. Risk of Bias assessment for Randomized Controlled Trials. [file 40798_2023_591_MOESM3_ESM.docx]

**Supplementary file 3 (S3)**

**Risk of bias assessment for randomized controlled trials**

| Study | Bias arising from randomization process | Bias arising from randomization process | Bias due to deviations from intended interventions (effect of adhering to intervention) | Bias due to missing outcome data | Bias in measurement of the outcome | Bias in selection of the reported result |
| --- | --- | --- | --- | --- | --- | --- |
| Akagi and Takahashi [42] | LOW | LOW | LOW | LOW | LOW | LOW |
| Andrade et al. [9] | LOW | LOW | LOW | LOW | LOW | LOW |
| Blazevich et al. [24] | LOW | LOW | LOW | LOW | LOW | LOW |
| Lima et al. [43] | HIGH | SOME CONCERNS | HIGH | LOW | LOW | LOW |
| Freitas and Mil-Homens [23] | SOME CONCERNS | LOW | LOW | LOW | LOW | LOW |
| Kay et al. [21] | SOME CONCERNS | LOW | SOME CONCERNS | LOW | LOW | LOW |
| Konrad and Tilp [26] | LOW | LOW | LOW | LOW | LOW | LOW |
| Longo et al. [16] | SOME CONCERNS | LOW | LOW | LOW | LOW | LOW |
| Moltubakk et al. [17] | LOW | LOW | LOW | LOW | LOW | LOW |
| Nakamura et al. [22] | SOME CONCERNS | LOW | LOW | LOW | LOW | LOW |
| Nakamura et al. [25] | SOME CONCERNS | LOW | SOME CONCERNS | LOW | LOW | LOW |
| Panidi et al. [15] | LOW | LOW | LOW | LOW | LOW | LOW |
| Şekir et al. [44] | LOW | LOW | LOW | LOW | LOW | LOW |
| Peixinho et al. [37] | SOME CONCERNS | LOW | LOW | LOW | LOW | LOW |
