# Supplementary material for: Muscle Architecture Adaptations to Static Stretching Training: A Systematic Review with Meta-Analysis
Source: Sports Med Open. 2023 Jun 15;9:47. doi: 10.1186/s40798-023-00591-7 (PMC10271914; doi:10.1186/s40798-023-00591-7)
Supplement: Supplementary file 4 — Additional file 4. Risk of Bias assessment for Controlled Trials. [file 40798_2023_591_MOESM4_ESM.docx]

**Supplementary file 4 (S4)**

**Risk of bias assessment for controlled trials**

| Study | Bias due to confounding | Bias in selection of participants into the study | Bias in classification of interventions | Bias due to deviations from intended interventions | Bias due to missing data | Bias in measurement of outcomes | Bias in selection of the reported result |
| --- | --- | --- | --- | --- | --- | --- | --- |
| Brusco et al. [41] | Low | Low | Low | Low | Low | Moderate | Low |
| Mizuno et al. [20] | Low | Low | Low | Low | Low | Moderate | Low |
| Yahata et al. [18] | Moderate | Low | Low | Low | Low | Moderate | Low |
| Simpson et al. [14] | Low | Moderate | Low | Low | Low | Moderate | Low |
| Warkene et al. [45] | Moderate | Moderate | Low | Low | Low | Moderate | Low |
