# Supplementary material for: Muscle Architecture Adaptations to Static Stretching Training: A Systematic Review with Meta-Analysis
Source: Sports Med Open. 2023 Jun 15;9:47. doi: 10.1186/s40798-023-00591-7 (PMC10271914; doi:10.1186/s40798-023-00591-7)
Supplement: Supplementary file 5 — Additional file 5. GRADE analysis. [file 40798_2023_591_MOESM5_ESM.docx]

**Supplementary file 5 (S5)**

**GRADE analysis**

|  | **Summary of findings table according to GRADE analysis** | | | | | | | **Evaluation components to lower quality** | | | | | | **Evaluation components to higher quality** | | |
| --- | --- | --- | --- | --- | --- | --- | --- | --- | --- | --- | --- | --- | --- | --- | --- | --- |
| **No** | **Outcome** | **Prevalence/risk ratio (95% CI)/Counts** | **Number of studies/entries** | **Number of Participants Exposure** | **Number of Participants Comparator** | **Quality of evidence (GRADE)** | **Comments** | Methodological design start point | Risk of bias | Inconsistencey of results | Indirectness | Imprecision | Publication bias | Large effect | Dose response | Confounding |
| 1 | Fascicle Length (by volume) STR vs CTR | 0.17(0.01-0.33) % | 14/30 | 459 | 440 | High ⨁⨁⨁⨁ | We are very confident that the true effect lies close to that of the estimate of the effect. | Randomized-trial studies: High quality | The included studies display >83% low risk of bias. No downgrade | Even though we used a random effect model meta-analysis, we consider heterogeneity as an index of inconsistency. I²=24%, low heterogeneity, <75%. No downgrade | Most of the studies display as a primary aim, a similar to the systematic review aim. No downgrade | The overall sample size is very large (n=899), therefore, it is unlikely to retrieve imprecise outcomes | We used a detailed but not exhaustive searching approach (i.e. grey literature and scientific organizations were not examined). No major funding from the industry. No major bias in the funnel plots. Downgrade 1 level | Z score >2.0, very large effect. Upgrade 1 level (2.03) | Dose response effect. Upgrade 1 level | We found no confounding factors that indicate upgrading |
| 2 | Fascicle Length (by intensity) STR vs CTR | 0.17(0.01-0.33) % | 14/30 | 459 | 440 | High ⨁⨁⨁⨁ | We are very confident that the true effect lies close to that of the estimate of the effect. | Randomized-trial studies: High quality | The included studies display >83% low risk of bias. No downgrade | Even though we used a random effect model meta-analysis, we consider heterogeneity as an index of inconsistency. I²=24%, low heterogeneity, <75%. No downgrade | Most of the studies display as a primary aim, a similar to the systematic review aim. No downgrade | The overall sample size is very large (n=899), therefore, it is unlikely to retrieve imprecise outcomes | We used a detailed but not exhaustive searching approach (i.e. grey literature and scientific organizations were not examined). No major funding from the industry. No major bias in the funnel plots. Downgrade 1 level | Z score >2.0, very large effect. Upgrade 1 level (2.03) | Dose response effect. Upgrade 1 level | We found no confounding factors that indicate upgrading |
| 3 | Fascicle Length (during stretching) STR vs CTR | 0.39(0.05-0.74) % | 6/9. | 165 | 154 | Moderate ⨁⨁⨁◯ | We are moderately confident in the effect estimate: The true effect is likely to be close to the estimate of the effect, but there is a possibility that it is substantially different | Randomized-trial studies: High quality | The included studies display >91% low risk of bias. No downgrade | Even though we used a random effect model meta-analysis, we consider heterogeneity as an index of inconsistency. I²=47%, low heterogeneity, <75%. No downgrade | Most of the studies display as a primary aim, a similar to the systematic review aim. No downgrade | The overall sample size is <800 (n=319), therefore, it is likely to retrieve imprecise outcomes. Downgrade 1 level | We used a detailed but not exhaustive searching approach (i.e. grey literature and scientific organisations were not examined). No major funding from the industry. No major bias in the funnel plots. Downgrade 1 level | Z score >2.0, very large effect. Upgrade 1 level (2.23) | NA | We found no confounding factors that indicate upgrading |
| 4 | Pennation Angle (by volume) STR vs CTR | 0.08(-0.07-0.22) % | 11/25. | 384 | 376 | Low ⨁⨁◯◯ | Our confidence in the effect estimate is limited: The true effect may be substantially different from the estimate of the effect. | Randomized-trial studies: High quality | The included studies display >83% low risk of bias. No downgrade | Even though we used a random effect model meta-analysis, we consider heterogeneity as an index of inconsistency. I²=0%, low heterogeneity. No downgrade | Most of the studies display as a primary aim, a similar to the systematic review aim. No downgrade | The overall sample size is <800 (n=760), therefore, it is likely to retrieve imprecise outcomes. Downgrade 1 level | We used a detailed but not exhaustive searching approach (i.e. grey literature and scientific organisations were not examined). No major funding from the industry. No major bias in the funnel plots. Downgrade 1 level | Z score between 0.6-1.2, moderate effect. No upagrade(1.03) | NA | We found no confounding factors that indicate upgrading |
| 5 | Pennation Angle (by intensity) STR vs CTR | 0.09 (-0.05-0.24) % | 11/25. | 384 | 376 | Very Low ⨁◯◯◯ | Our confidence in the effect estimate is limited: The true effect may be substantially different from the estimate of the effect. | Randomized-trial studies: High quality | The included studies display >83% low risk of bias. No downgrade | Even though we used a random effect model meta-analysis, we consider heterogeneity as an index of inconsistency. I²=0%, low heterogeneity. No downgrade | Most of the studies display as a primary aim, a similar to the systematic review aim. No downgrade | The overall sample size is <800 (n=760), therefore, it is likely to retrieve imprecise outcomes. Downgrade 1 level | We used a detailed but not exhaustive searching approach (i.e. grey literature and scientific organizations were not examined). No major funding from the industry. No major bias in the funnel plots. Downgrade 1 level | Z score between 1.2-2, large effect. Upgrade 1 level (1.28) | NA | We found no confounding factors that indicate upgrading |
| 6 | Muscle Thickness (by volume) STR vs CTR | 0.11 (-0.05-0.28) % | 15/31 | 481 | 466 | Moderate ⨁⨁⨁◯ | We are very confident that the true effect lies close to that of the estimate of the effect. | Randomized-trial studies: High quality | The included studies display >82% low risk of bias. No downgrade | Even though we used a random effect model meta-analysis, we consider heterogeneity as an index of inconsistency. I²=33%, low heterogeneity. No downgrade | Most of the studies display as a primary aim, a similar to the systematic review aim. No downgrade | The overall sample size is very large (n=947), therefore, it is unlikely to retrieve imprecise outcomes | We used a detailed but not exhaustive searching approach (i.e. grey literature and scientific organisations were not examined). No major funding from the industry. No major bias in the funnel plots. Downgrade 1 level | Z score between 1.2-2, large effect. Upgrade 1 level (1.35) | NA | We found no confounding factors that indicate upgrading |
| 7 | Muscle Thickness (by intensity) STR vs CTR | 0.11 (-0.05-0.28) % | 15/31 | 481 | 466 | High ⨁⨁⨁⨁ | We are very confident that the true effect lies close to that of the estimate of the effect. | Randomized-trial studies: High quality | The included studies display >82% low risk of bias. No downgrade | Even though we used a random effect model meta-analysis, we consider heterogeneity as an index of inconsistency. I²=33%, low heterogeneity. No downgrade | Most of the studies display as a primary aim, a similar to the systematic review aim. No downgrade | The overall sample size is very large (n=947), therefore, it is unlikely to retrieve imprecise outcomes | We used a detailed but not exhaustive searching approach (i.e. grey literature and scientific organisations were not examined). No major funding from the industry. No major bias in the funnel plots. Downgrade 1 level | Z score between 1.2-2, large effect. Upgrade 1 level (1.35) | Dose response effect. Upgrade 1 level | We found no confounding factors that indicate upgrading |

**Note**: NA: not applicable; STR: Stretching Group; CTR: Control Group
