# Supplementary figures and images for: Muscle Architecture Adaptations to Static Stretching Training: A Systematic Review with Meta-Analysis
Source: Sports Med Open. 2023 Jun 15;9:47. doi: 10.1186/s40798-023-00591-7 (PMC10271914; doi:10.1186/s40798-023-00591-7)

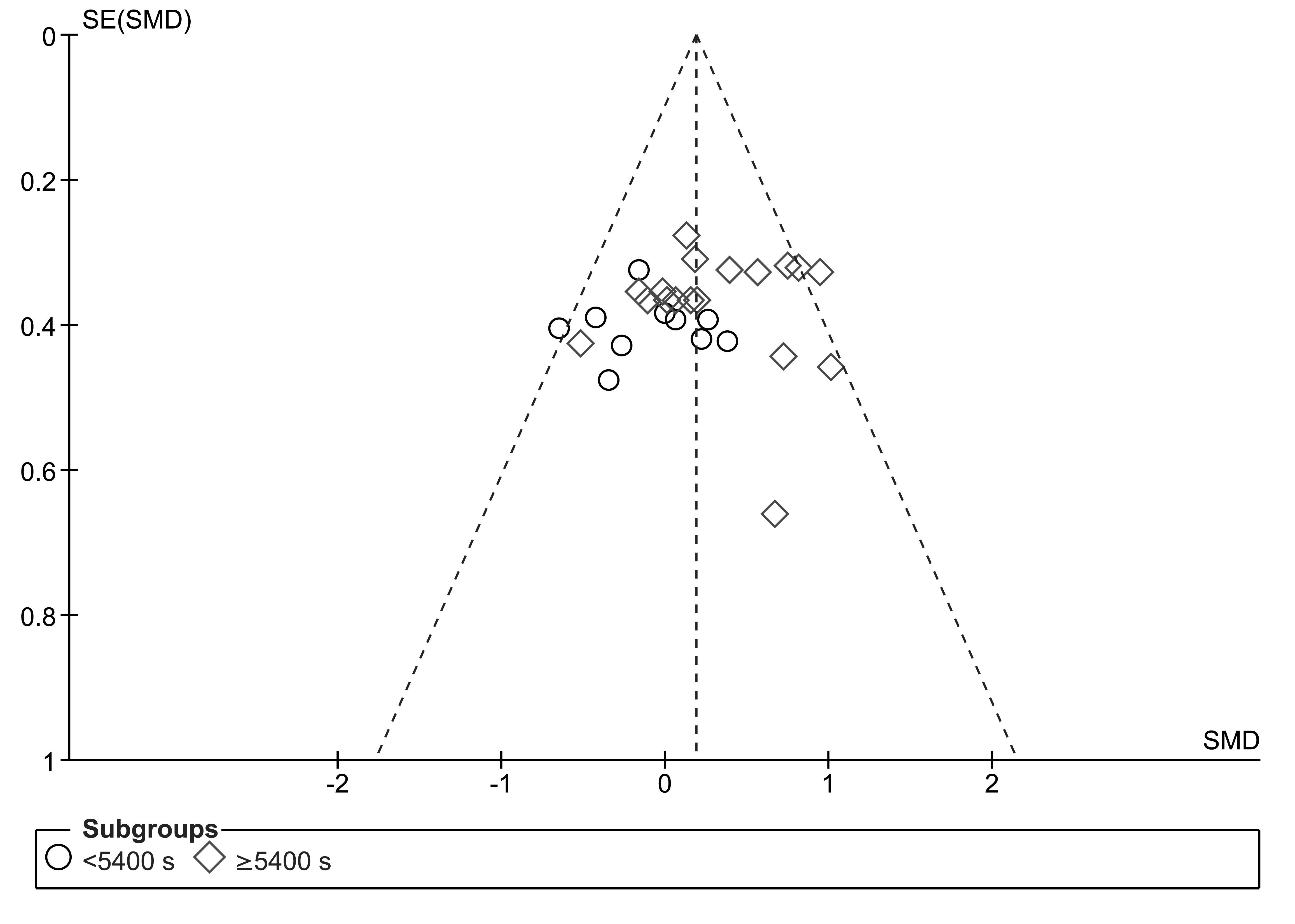

Supplement: Supplementary file 6 — Additional file 6: Fig. 1. Funnel plot for fascicle length. [file 40798_2023_591_MOESM6_ESM.tif]

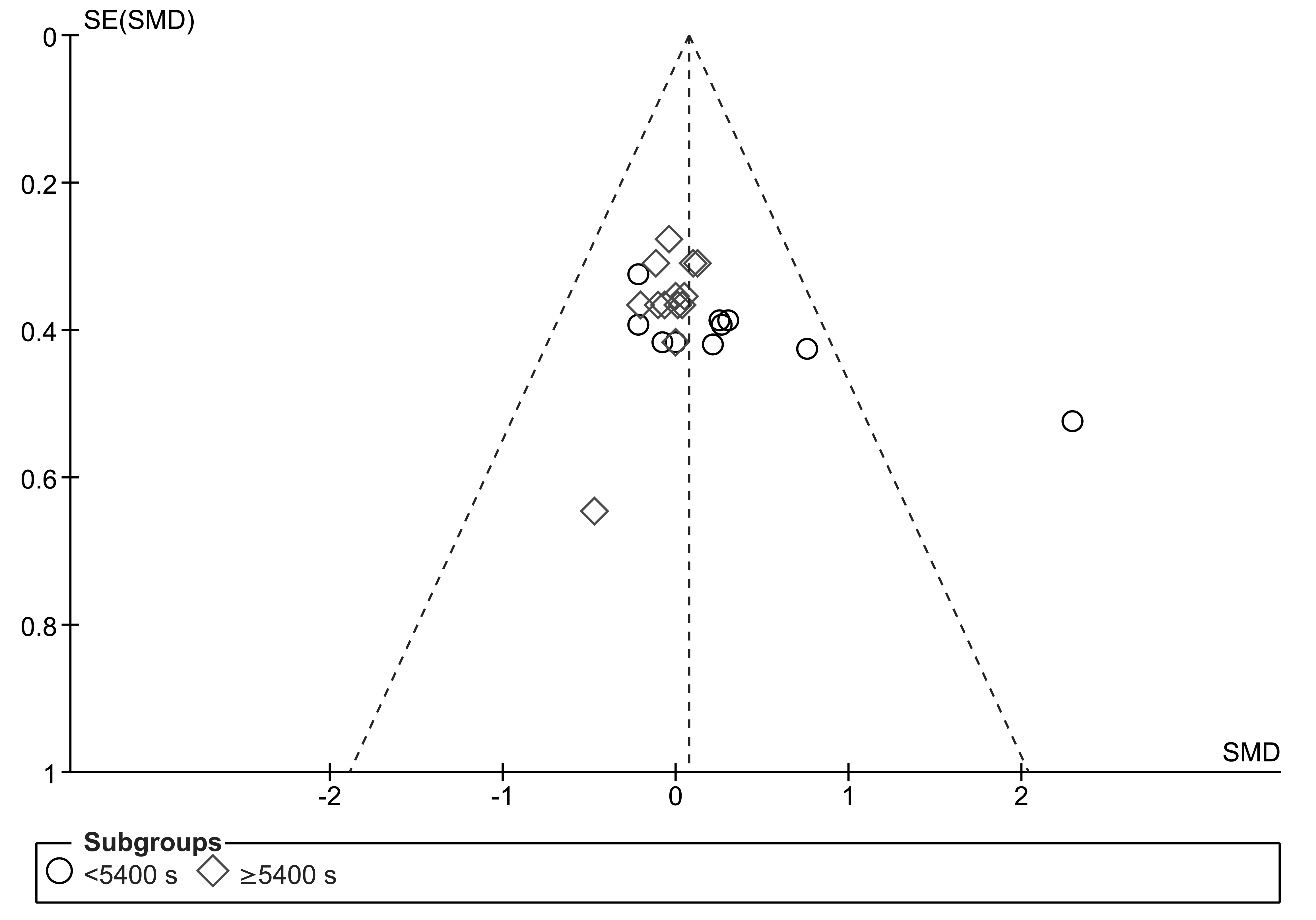

Supplement: Supplementary file 7 — Additional file 7: Fig. 2. Funnel plot for fascicle angle. [file 40798_2023_591_MOESM7_ESM.tif]

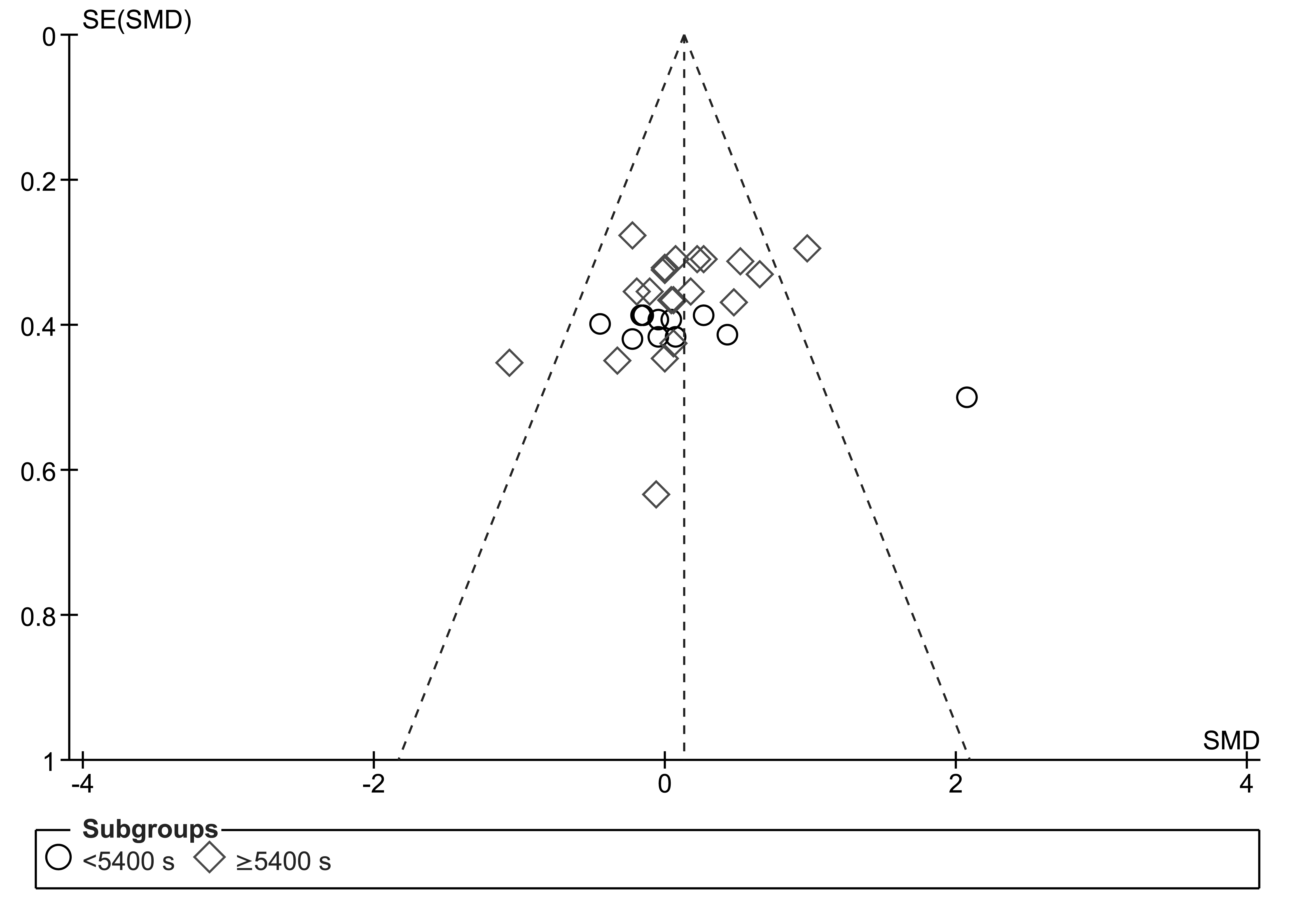

Supplement: Supplementary file 8 — Additional file 8: Fig. 3. Funnel plot for muscle thickness. [file 40798_2023_591_MOESM8_ESM.tif]
